# Supplementary material for: Secondary Dengue Infection Elicits Earlier Elevations in IL-6 and IL-10 Levels
Source: Int J Mol Sci. 2024 Oct 19;25(20):11238. doi: 10.3390/ijms252011238 (PMC11508614; doi:10.3390/ijms252011238)
Supplement: Supplementary file 1 [file ijms-25-11238-s001.zip › ijms-3140556-supplementary.pdf]

Supplementary Table S1:

Cytokine levels and clinical parameters during phase II of primary infections (*n*=27).

**Characteristics**	**IL-6***	***p***	**IL-10***	***p***
WBC 5000/ μL				
<	6.4 (3.4-21.0)	0.049	30.3 (11.2-139.3)	0.873
>	21.9 (7.5-55.4)		34.5 (15.4-77.7)	
Platelets 100,000/μL				
<	53.3 (8.3-276.9)	0.070	13.5 (12.2-53.5)	0.355
>	6.6 (3.8-23.9)		33.2 (11.0-118.0)	
AST ULN (U/L) #				
<	15.7 (4.0-71.0)	0.216	93.9 (9.5-143.5)	0.364
>	6.6 (3.7-10.9)		20.5 (10.1-47.5)	
ALT ULN (U/L) #				
<	6.9 (4.3-12.9)	0.727	22.6 (10.5-110.9)	0.662
>	10.3 (3.5-58.6)		36.1 (10.7-142.2)	

*Median (interquartile range), WBC: White blood cells, ULN: upper limit of normal (>40 U/L),

#Available in 20 patients. Median values were compared using the Mann-Whitney U test.
